# Neuromorphic electronic tactile system for human‐level tactile feedback

**DOI:** 10.1002/ctm2.70413

**Published:** 2025-07-10

**Authors:** Zhi‐Bin Zhang, Libo Chen

**Affiliations:** ^1^ Department of Electrical Engineering Solid State Electronics Uppsala University Uppsala Sweden

**Keywords:** electronic skin, neuromorphic systems, spiking neural networks, tactile classification

1

Tactile perception is fundamental for human interaction with the environment.^1^ Through the use of hands and fingertips, individuals can explore and discern the properties of objects. Additionally, tactile feedback is essential for the dexterous handling of tools, allowing for rapid adjustments in the motor system. This perception arises from the integration of multiple types of mechanoreceptors embedded in the skin, the central pathways that transmit tactile information to the brain, and cognitive processes in the brain.^2^ Damage to the skin, nerve pathways, or brain can result in various disabilities that significantly impair a patient's quality of life.

In this context, developing effective and advanced artificial tactile feedback systems is crucial for clinical applications, particularly those aimed at restoring functions lost due to nervous system injuries.^3^ Neuroprosthetic systems that provide adequate tactile feedback can be tailored to enhance proper walking and the dexterous manipulation of objects with the hands and fingers. For amputees, tactile feedback enhances control over bionic hands and fosters a greater sense of ownership. Additionally, stimulating the remaining peripheral sensory nerves with tactile feedback can effectively alleviate phantom pain, which often afflicts patients who have lost limbs, such as in car accidents. Furthermore, imparting tactile sensing capabilities to artificial skin can be highly beneficial for patients with burn injuries, offering a functional replacement for damaged skin.

To create tactile feedback, electronic skins (e‐skins) have been developed. e‐Skins are large‐area, flexible electronic devices that consist of tactile sensor arrays.^4^ As illustrated in Figure 1A, these arrays transduce mechanical stimuli (e.g. pressure) into analogue signals, such as voltage or current. Traditionally, an electronic circuit connected to the e‐skin reads these analogue signals by sequentially and periodically sampling the individual sensors. In the past years, research on tactile feedback has primarily focused on the development of materials and the exploration of device structures to enhance performance in mechano‐electric transduction. More recently, machine learning techniques, particularly artificial neural networks, have been employed for object classification based on grasp patterns.^5^ However, this conventional approach presents several significant limitations that pose serious concerns for real‐world applications.

**FIGURE 1:**
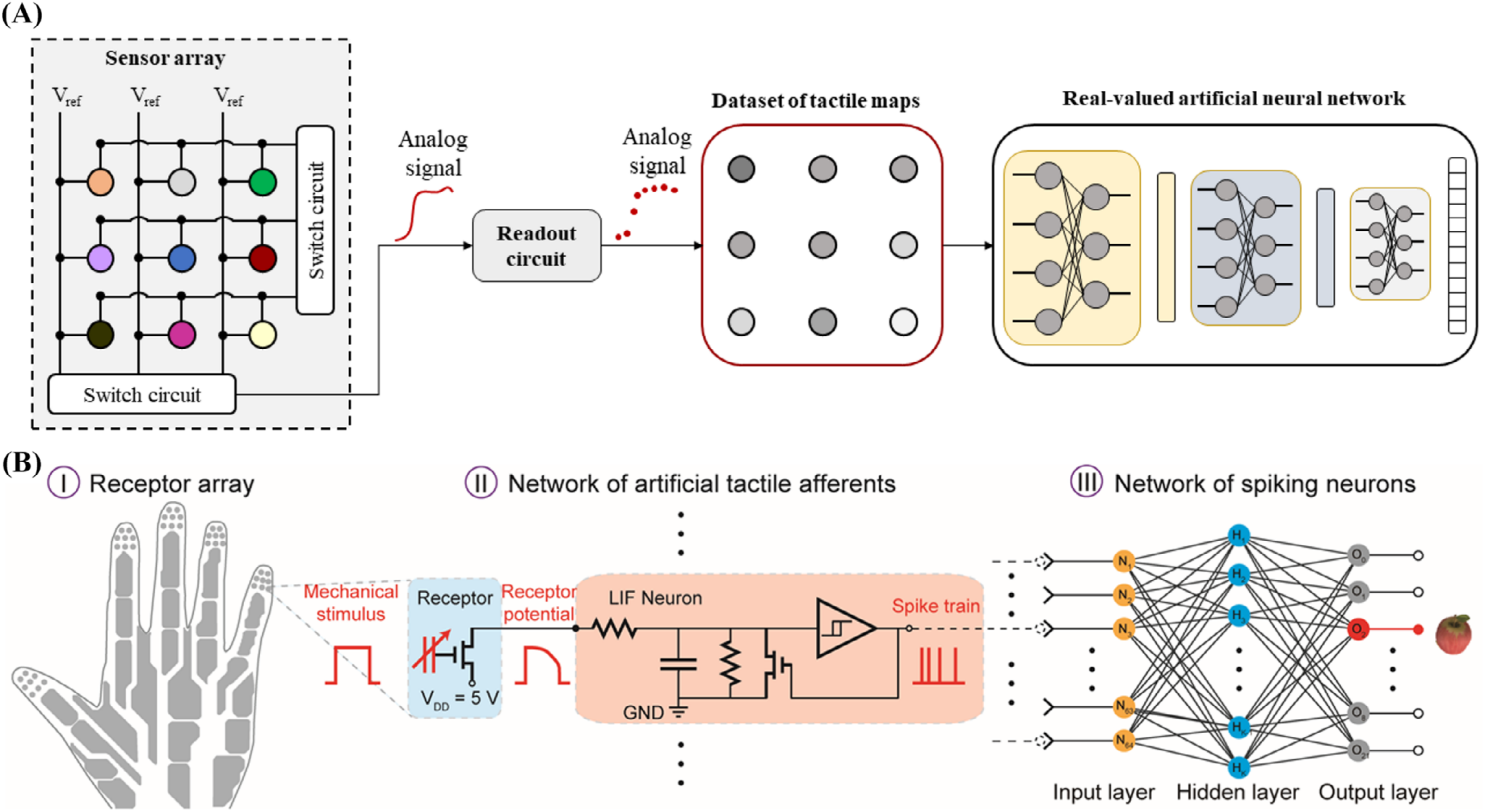
(A) The existing e‐skin features a sensor array whose analogue signals are read out serially, forming a dataset of tactile maps which can be processed by using real‐valued artificial neural networks. (B) The neuromorphic tactile system,^7^ as representing a tactile spiking neural network (t‐SNN), comprises (I) a receptor array, (II) a neuromorphic circuit of artificial neurons and (III) a software‐implemented network of spiking neurons. Figure B adapted [^7^].

First, there is no local signal processing, which prevents current e‐skins from providing human‐like tactile feedback. Secondly, the serial readout mechanism results in a resolvable time difference of approximately 100 ms. This resolvable time difference is more than 10 times longer than that of the human nervous system,^6^ making it inadequate for handling rapidly changing tactile events required for precise and quick motor control. While increasing the sampling speed and data rates could reduce this time delay, it would also increase power consumption. Thirdly, current e‐skins and conventional readout circuits generally suffer from high power consumption, even in the absence of stimulation. This issue becomes more severe as the number of sensors increases, particularly when handling highly dynamic tactile events. Consequently, this limits the scalability and practical application of e‐skins in real‐world scenarios.

To advance tactile feedback technology, our lab has recently developed a neuromorphic tactile system capable of rapid object recognition through finger touch and hand grasp.^7^ This system represents a three‐layer tactile spiking neural network (t‐SNN), loosely emulating the somatosensory pathway that transmits tactile information from the skin to the brain (Figure 1B). It comprehensively employs spikes (action potentials) for signal processing and computation.

The t‐SNN was implemented using a combination of hardware and software. There are 64 neuromorphic tactile sensors, each comprising an artificial receptor and an electronic circuit that mimics a sensory neuron. The artificial receptors are based on triboelectric nanogenerators, which harness mechanical energy during tactile sensing, making them self‐powered. These receptors were designed to match the areas of the receptive fields on a human hand. Upon contact with an object, the artificial receptors capture tactile stimuli and convert them into analogue electrical signals. The artificial neurons then convert these analogue signals into spikes using a “leaky‐integrate‐fire” model.

When the e‐skin touches and grasps objects, multiple tactile sensors are activated in a specific sequence, resulting in an ensemble of spike trains. This encoding method achieves a resolvable time difference of around 1 ms, comparable to the biological nervous system. The software component of the system was trained using surrogate descent method with backpropagation, optimising the network structure to extract dynamic information about the surfaces touched and objects grasped. This neuromorphic approach enables precise object recognition before the completion of grasp actions. The system demonstrated effective classification of material's properties of 16 textures by touch and 22 daily life items by grasp. Moreover, it is typically driven by tactile events, consuming negligible power in idle states. The neuromorphic design approach also ensures robust performance against uncertainties and sensor damage.

These results offer promising opportunities to enhance neuroprosthetic systems for individuals suffering from conditions such as stroke and paralysis. Stroke, caused by cell death in the brain, impairs the function of neural circuits in the affected area. Currently, stroke poses a significant threat, with 25% of the global population at risk of experiencing a stroke in their lifetime.^8^ Stroke adversely affects motor control, degrading abilities such as walking and fine motor skills necessary for manipulating objects with hands and fingers. Additionally, stroke often leads to paralysis, which is particularly caused by damage to the spinal cord and is frequently accompanied by sensory loss in the affected regions. The advancements in the neuromorphic tactile system have the potential to provide high‐level tactile feedback to neuroprosthetic systems. These systems aim to restore tactile sensation and grasping functions in individuals with sensory and motor disabilities, thereby significantly improving their quality of life.^9^

Despite the great potential applications in clinical and homecare settings, the sophisticated tactile system faces challenges.^9^ The primary challenge is the limited selectivity of the neural interface when using the electrical output to stimulate the cortex.^10^ Another significant challenge is the substantial variability between patients. Therefore, further development is needed to incorporate additional neural processing steps and encoding strategies to facilitate effective electrical stimulation. Moreover, comprehensive means, including psychophysical tests, biophysical models, and machine learning methods, may be necessary to optimise and personalise tactile sensory feedback. This research will help to ensure that tactile feedback is effectively and appropriately tailored to individual needs, maximising the benefits of neuroprosthetic systems.

## AUTHOR CONTRIBUTIONS

Zhang wrote the manuscript. Zhang and Chen improved the manuscript.

## CONFLICT OF INTEREST STATEMENT

The authors declare no conflict of interest.

## ETHICS STATEMENT

This work does not involve animals, human, and other ethical issues.
